# Effects of Propranolol on Growth, Lipids and Energy Metabolism and Oxidative Stress Response of *Phaeodactylum tricornutum*

**DOI:** 10.3390/biology9120478

**Published:** 2020-12-18

**Authors:** Bernardo Duarte, Eduardo Feijão, Ricardo Cruz de Carvalho, Irina A. Duarte, Marisa Silva, Ana Rita Matos, Maria Teresa Cabrita, Sara C. Novais, Marco F. L. Lemos, João Carlos Marques, Isabel Caçador, Patrick Reis-Santos, Vanessa F. Fonseca

**Affiliations:** 1 MARE—Marine and Environmental Sciences Centre, Faculdade de Ciências da Universidade de Lisboa, Campo Grande, 1749-016 Lisbon, Portugal; eduardomof@gmail.com (E.F.); rfcruz@fc.ul.pt (R.C.d.C.); iaduarte@fc.ul.pt (I.A.D.); mpdsilva@fc.ul.pt (M.S.); micacador@fc.ul.pt (I.C.); pnsantos@fc.ul.pt (P.R.-S.); vffonseca@fc.ul.pt (V.F.F.); 2 Departamento de Biologia Vegetal, Faculdade de Ciências da Universidade de Lisboa, Campo Grande, 1749-016 Lisbon, Portugal; armatos@fc.ul.pt; 3 cE3c, Centre for Ecology, Evolution and Environmental Changes, Faculdade de Ciências, Universidade de Lisboa, Campo Grande, Edifício C2, Piso 5, 1749-016 Lisbon, Portugal; 4 BioISI—Biosystems and Integrative Sciences Institute, Plant Functional Genomics Group, Departamento de Biologia Vegetal, Faculdade de Ciências da Universidade de Lisboa, Campo Grande, 1749-016 Lisbon, Portugal; 5 Centro de Estudos Geográficos (CEG), Instituto de Geografia e Ordenamento do Território (IGOT), Universidade de Lisboa, Rua Branca Edmée Marques, 1600-276 Lisbon, Portugal; tcabrita@campus.ul.pt; 6 MARE—Marine and Environmental Sciences Centre, ESTM, Politécnico de Leiria, 2520-641 Peniche, Portugal; sara.novais@ipleiria.pt (S.C.N.); marco.lemos@ipleiria.pt (M.F.L.L.); 7 MARE—Marine and Environmental Sciences Centre, Department of Life Sciences, University of Coimbra, 3000 Coimbra, Portugal; jcmimar@ci.uc.pt; 8 Southern Seas Ecology Laboratories, School of Biological Sciences, The University of Adelaide, Aldeide, SA 5005, Australia

**Keywords:** energy metabolism, pharmatoxicology, photobiology, primary producers, toxicophenomics

## Abstract

**Simple Summary:**

In the past two decades, increasing attention has been directed to investigate the incidence and consequences of pharmaceuticals in the aquatic environment. Propranolol is a non-selective β-adrenoceptor blocker used in large quantities worldwide to treat cardiovascular conditions. Diatoms (model organism) exposed to this compound showed evident signs of oxidative stress, a significant reduction of the autotrophic O_2_ production and an increase in the heterotrophic mitochondrial respiration. Additionally, diatoms exposed to propranolol showed a consumption of its storage lipids. In ecological terms this will have cascading impacts in the marine trophic webs, where these organisms are key elements, through a reduction of the water column oxygenation and essential fatty acid availability to the heterotrophic organisms that depend on these primary producers. In ecotoxicological terms, diatoms photochemical and fatty acid traits showed to be potential good biomarkers for toxicity assessment of diatoms exposed to this widespread pharmaceutical compound.

**Abstract:**

Present demographic trends suggest a rise in the contributions of human pharmaceuticals into coastal ecosystems, underpinning an increasing demand to evaluate the ecotoxicological effects and implications of drug residues in marine risk assessments. Propranolol, a non-selective β-adrenoceptor blocker, is used worldwide to treat high blood pressure conditions and other related cardiovascular conditions. Although diatoms lack β-adrenoceptors, this microalgal group presents receptor-like kinases and proteins with a functional analogy to the animal receptors and that can be targeted by propranolol. In the present work, the authors evaluated the effect of this non-selective β-adrenoceptor blocker in diatom cells using *P. tricornutum* as a model organism, to evaluate the potential effect of this compound in cell physiology (growth, lipids and energy metabolism and oxidative stress) and its potential relevance for marine ecosystems. Propranolol exposure leads to a significant reduction in diatom cell growth, more evident in the highest concentrations tested. This is likely due to the observed impairment of the main primary photochemistry processes and the enhancement of the mitochondrial respiratory activity. More specifically, propranolol decreased the energy transduction from photosystem II (PSII) to the electron transport chain, leading to an increase in oxidative stress levels. Cells exposed to propranolol also exhibited high-dissipated energy flux, indicating that this excessive energy is efficiently diverted, to some extent, from the photosystems, acting to prevent irreversible photoinhibition. As energy production is impaired at the PSII donor side, preventing energy production through the electron transport chain, diatoms appear to be consuming storage lipids as an energy backup system, to maintain essential cellular functions. This consumption will be attained by an increase in respiratory activity. Considering the primary oxygen production and consumption pathways, propranolol showed a significant reduction of the autotrophic O_2_ production and an increase in the heterotrophic mitochondrial respiration. Both mechanisms can have negative effects on marine trophic webs, due to a decrease in the energetic input from marine primary producers and a simultaneous oxygen production decrease for heterotrophic species. In ecotoxicological terms, bio-optical and fatty acid data appear as highly efficient tools for ecotoxicity assessment, with an overall high degree of classification when these traits are used to build a toxicological profile, instead of individually assessed.

## 1. Introduction

Over 2.3 billion people live near the sea, with 21 of the world’s megacities located in coastal areas [1,2]. This anthropogenic pressure has inevitable impacts on marine coastal ecosystems [3]. In the past two decades, increasing attention has been directed to investigate the incidence and consequences of pharmaceuticals in the aquatic environment, mainly in freshwater ecosystems [4]. However, the impacts of these compounds in the marine realm has received considerably less attention [4]. Nevertheless, demographic trends suggest an increase of human pharmaceutical inputs into coastal environments, highlighting the need to evaluate probable exposure scenarios and repercussions of drug residues for coastal and marine hazard assessments [4,5]. According to the World Health Organization, pharmaceuticals concentrations in aquatic systems are expected to increase due to the increased availability for a growing global population [6]. Moreover, the release of pharmaceuticals into marine environments at levels high enough to produce biological impacts may act as an extra stressor on these environments already impacted by global changes, eutrophication and overfishing [7]. Pharmaceuticals act differently from classical contaminants, as they are intended to be bioactive at reduced concentrations [8]. Moreover, these molecules are designed to act on specific target receptors, some of which are widely dispersed and present in several non-mammal and aquatic organisms, that during evolution remained conserved. Therefore, even at low concentrations, these contaminants represent a threat, with additional concerns due to their unknown modes of action, depending on the interaction pathways and potentially unidentified cross-talk mechanisms triggered in aquatic organisms [8].

Propranolol, a non-selective β-adrenoceptor blocker, is produced, prescribed and sold in large quantities worldwide to treat high blood pressure conditions and other related cardiovascular diseases [9,10,11]. Propranolol is fairly persistent [9], bioaccumulative [12] and highly water-soluble with a low degree of volatility [13], and presents a low tendency for organic matter adsorption [14]. Considering these hydrophilic features, propranolol can, therefore, remain in the aquatic phase after wastewater treatment [15]. The environmental propranolol concentrations range from 56 in estuaries [16] to 590 ng L^−1^ in rivers [17], 1900 ng L^−1^ in wastewater effluents [18] and 6500 ng L^−1^ in hospital effluents [19,20]. Due to resemblances between vertebrate animals and human β-adrenergic receptors, effects of propranolol exposure in aquatic vertebrate biota are predictable [21]. However, other groups of organisms (e.g., plants and algae) have distinct but functionally similar β-adrenergic receptors, which can also be targeted by non-specific β-adrenoceptor blockers [22]. In particular, there are a variety of biochemically similar receptor-like kinases (RLK) that can be targeted by this pharmaceutical in the plant kingdom [22]. In phototrophic organisms, these receptors participate in a wide array of processes, such as sensory mechanisms and innate immune responses, and developmental processes and reactive oxygen species (ROS) regulation [23]. Nevertheless, the information is scarce on the possible effects of propranolol in marine phototrophs and its impacts on their physiology and ecological roles.

Diatoms are one of the most abundant groups of microalgae, forming the base of the marine and estuarine food webs worldwide [24], and are responsible for about 20% of the global primary productivity [25]. Thus, diatoms are a main marine carbon sink and essential oxygen-production agents, essential to supporting marine heterotrophic life [26]. These organisms produce essential fatty acids (EFAs), such as the omega 6 (ɷ-6) linoleic acid and the omega 3 (ɷ-3) linolenic acid, precursors for long-chain polyunsaturated fatty acids (LC-PUFAs), such as eicosapentaenoic acid (EPA) and docosahexaenoic acid (DHA). These LC-PUFAs are essential molecules for the maintenance of cardiovascular functions, immune and inflammatory reactions and neurological tissue arrangement in heterotrophic species [27]. EFAs are diet-acquired since heterotrophs have limited ability to produce them [28,29]. Due to its cosmopolitan distribution [30], fully sequenced genome [31] and role as a biomonitor species able to reflect early signs of stress [32], *Phaeodactylum tricornutum*, is a marine diatom frequently used in stress biology and ecotoxicological studies (e.g., temperature [33,34], heavy metal exposure [30,35], nutrient depletion [36] or emerging pollutants [37,38])). Common physiological and biochemical traits evaluated in these studies involve photochemical feedbacks [30,37], oxidative stress responses [38] and variations in membrane fatty acids content and unsaturation [33,36,37]. All these features have the potential to be used as in vitro ecotoxicological biomarkers, essential to disentangle the mechanisms of action of emerging contaminants, namely human pharmaceuticals. The application of toxicophenomic techniques (e.g., application of phenotyping techniques such as chlorophyll fluorescence analysis in ecotoxicological studies), in particular of high-throughput bio-optical methods, allows the acquisition of high volumes of physiological data over time, without organism sacrifice, and assay disturbance or interference [30,37]. The combined use of non-invasive phenotyping techniques and classical biochemical tools have proven to be an efficient approach in *P. tricornutum* ecotoxicological studies, providing new insights into cellular impacts and mode of action of emerging and classical contaminants [30,37].

Considering this, the present work aims to evaluate the impacts of diatom exposure to the non-selective β-adrenoceptor blocker propranolol. For this *P. tricornutum* growth, energetic and fatty acid metabolism and oxidative stress will be addressed to unravel this compound’s mode of action in diatoms.

## 2. Materials and Methods

### 2.1. Experimental Setup

*Phaeodactylum tricornutum* Bohlin (Bacillariophyceae; strain IO 108–01, Instituto Português do Mar e da Atmosfera (IPMA)) axenic cell cultures (maintained under asexual reproduction conditions) were placed to grow in f/2 medium [39], under constant aeration in a phytoclimatic chamber, at 18 °C, programmed with a 14/10 h day/night photoperiod (RGB 1:1:1, maximum PAR 80 μmol photons m^−2^ s^−1^), a sinusoidal function to mimic sunrise and sunset, and light intensity at noon, set to replicate a natural light environment [33]. Cultures are periodically inspected visually under the microscope to ensure their axenic state. Exposure trials were conducted according to the Organization for Economic Cooperation and Development (OECD) recommendations for algae assays [40], with minor adaptations, and the suggested initial cell density for microalgae cells with comparable dimensions to *P. tricornutum* (initial cell density = 2.7 × 10^5^ cells mL^−1^). According to OECD guidelines, carbon was provided to the cultures through aeration with ambient air. Exposure time was reduced to 48 h since in previous studies was observed that after 72 h the cultures enter the stationary phase and thus exhibit aging effects that can mask the exposure trial [33]. Forty-eight hours after inoculation, cells were exposed to 0, 0.3, 8, 80, 150 and 300 μg L^−1^ propranolol. Three independent 250 mL batch cultures (replicates) were considered for all treatments. Exposure occurred for 48 h to ensure it occurred during the exponential growth phase [30,33,37]. Target concentrations were selected aiming to cover a concentration gradient reflecting not only the detected environmental concentrations found in the literature and but concentrations known to have significant biological effects in *P. tricornutum* (EC_50_ = 252–329 μg L^−1^ propranolol according to Franzellitti et al., 2015) [20,41]. As observed in precious works [30,33,37] this is a fast growing strain and thus the exposure period was reduced from 72 to 48 h to avoid cell ageing processes that possibly occur during the stationary phase beyond the 48 h timepoint. All manipulations were executed within a laminar flow hood chamber, ensuring standard aseptic conditions.

### 2.2. Diatom Cell Density Measurements and Pellet Collection

*Phaeodactylum tricornutum* cells (1 mL volume sample) were counted using a Neubauer improved counting chamber, coupled with an Olympus BX50 (Tokyo, Japan) inverted microscope, at 400-times magnification. According to [42], diatom growth was calculated using the mean specific growth rate per day, computed from the difference between initial and final logarithmic cell densities along the exposure period. Growth inhibition concentration (IC_50_) was calculated according to the OECD guidelines for the algae inhibition test [40]. Shortly, the average specific growth rates at different propranolol exposure concentrations were used to estimate the concentration causing a 50% reduction in the growth rate and hereafter expressed as the IC_50_ [40]. After 48 h of exposure, cells were harvested for biochemical analysis by centrifugation at 4000× *g* for 15 min at 4 °C and the pellets frozen in liquid nitrogen and stored at −80 °C. Three biological replicates for all tested conditions were considered for each analysis and collected from a total of 18 experimental units.

### 2.3. Chlorophyll a Pulse Amplitude Modulated Fluorometry

Before cell harvesting, 1 mL of each replicate culture was used for bio-optical assessment, using chlorophyll-a pulse amplitude modulated (PAM) fluorometry (FluorPen FP100, Photo System Instruments, Brno, Czech Republic). Cell subsamples for bio-optical assessment were acclimated for 15 min in the dark and chlorophyll transient light curves were generated using the preprogrammed OJIP protocol, according to [37]. The parameters determined and calculated by the software from this analysis are shown in Table 1 [43,44].

### 2.4. Cell Energy Allocation and Mitochondrial Metabolism

Cell pellets were disrupted by ultrasonication in 1 mL of ultra-pure water. Aliquots of the resulting homogenates were used to evaluate lipid, carbohydrate and protein contents, and the activity of the electron transport system (ETS). Milli-Q water was used as a reaction blank in all assays. All measurements were made by spectrophotometric means, at 25 °C, in a synergy H1 hybrid multimode microplate reader (Biotek^®^ Instrument, Winooski, VT, USA). Total lipids, proteins and carbohydrates extraction and analysis were performed according to De Coen and Janssen (1997), with slight modifications [45]. For available energy (Ea) determination, the total protein, carbohydrates and lipids content were converted into energetic equivalents, by using the corresponding combustion energy (17,500 mJ mg^−1^ carbohydrates, 24,000 mJ mg^−1^ protein and 39,500 mJ mg^−1^ lipid) [46]. The mitochondrial electron transport system (ETS) activity was determined according to [47] with the modifications described by [48]. Based on the theoretical stoichiometrical relationship that for each 2 μmol of INT-formazan formed, 1 μmol of O_2_ was consumed in the electron transport system and the cellular energy consumption (Ec) was calculated using the ETS results. The computed oxygen consumption was converted using the specific oxyenthalpic equivalents for an average lipid, protein and carbohydrate mixture of 480 kJ mol^−1^ O_2_ into energetic equivalents [46]. Cellular energy allocation (CEA) values, standardized to 10^6^ cells, were determined based on lipid, carbohydrate and protein content measurements and ETS activity for each sample [49]:

(1) $$\mathrm{CEA}=\frac{\mathrm{Ea}}{\mathrm{Ec}}$$

where,

(2) $$\mathrm{Ea}=\mathrm{carbohydrate}+\mathrm{lipid}+\mathrm{protein}\;\left(\mathrm{mJ}\;10^{-6}\mathrm{cells}\right)$$

(3) $$\mathrm{Ec}=\mathrm{ETS}\;\mathrm{activity}\;\left(\mathrm{mJ}\;10^{-6}\;\mathrm{cells}\right)$$

### 2.5. Oxidative Stress

Soluble protein was determined from cell pellets with 1 mL of 50 mM sodium phosphate buffer (pH 7.6) with 0.1 mM Na-EDTA, followed by sonication for 1 min. Samples were centrifuged at 10,000× *g* for 10 min at 4 °C, and the supernatant was collected. Protein content was determined according to Bradford (1976). Catalase (CAT), ascorbate peroxidase (APx) and superoxide dismutase (SOD) activities were assayed by spectrophotometric means using specific substrates as previously described [50,51,52,53]. Lipid peroxidation products were analyzed spectrophotometrically [54], using trichloroacetic acid extraction before the reaction with thiobarbituric acid. Results were expressed as malondialdehyde (MDA) equivalents, calculated as in [33].

### 2.6. Fatty Acids Profile

Cell pellets were submitted to direct transesterification with daily prepared methanol sulfuric acid (97.5:2.5, v/v) at 70 °C for 60 min [55]. Subsequently, fatty acids methyl esters (FAMEs) were recovered using petroleum ether and the solvent evaporated under a N_2_ flow in a dry bath at 30 °C [33,37]. FAMEs were resuspended in hexane and 1 µL was injected in a gas chromatograph (Varian 430-GC gas chromatograph, Middelburg, The Netherlands), equipped with a hydrogen flame ionization detector set at 300 °C. The temperature of the injector was set to 270 °C, with a split ratio of 50. The fused-silica capillary column (50 m × 0.25 mm; WCOT Fused Silica, CP-Sil 88 for FAME; Varian, Middelburg, The Netherlands) was maintained at a constant N_2_ flow of 2.0 mL min^−1^ and the oven set at 190 °C. Fatty acids identification was achieved by comparison of retention times with standards (Sigma-Aldrich, St. Louis, MO, USA), and chromatograms analyzed by the peak surface method, using the Galaxy software. The internal standard used was pentadecanoic acid (C15:0). The double bond index (DBI), a characteristic indicator of membrane unsaturation levels [33] was calculated as follows:

(4) $$\mathrm{DBI}=\frac{\%\mathrm{monoenes}+2\times \%\mathrm{dienes}+3\times \%\mathrm{trienes}+4\times \%\mathrm{tetraenes}+5\times \%\mathrm{pentaenes}}{100}$$

### 2.7. Statistical Analysis

Owing to the absence of normality and homogeneity of variances of our data, pairwise comparisons between different sample groups were assessed through non-parametric Kruskal–Wallis tests. Spearman correlation tests were performed to evaluate if there was a dose–response behavior between the exogenous propranolol concentrations tested and the growth, photochemical and biochemical variables. Both Kruskal–Wallis and Spearman tests were performed using Statistica software (StataSoft, version 12.5.192.7). Statistical significance was considered at *p* < 0.05. A multivariate approach was employed to test for variations in the complete photochemical and fatty acid metabolism [37,56,57]. Canonical analysis of principle (CAP) coordinates, using Euclidean distances, were preformed to plot in a canonical space the dissimilarities regarding fatty acids and photochemical studied variables while preforming a cross-validation step and determining the allocation efficiency into the different treatment groups. This multivariate methodology is unaffected by heterogeneous data and frequently used to compare different sample assemblies using the inherent features of each assembly (metabolic traits) [30,37,56,58]. Multivariate statistical analyses were performed using Primer 6 software (version 6.1.13, Plymouth, UK) [59].

## 3. Results

### 3.1. Growth-Related Features

**Figure 1:**
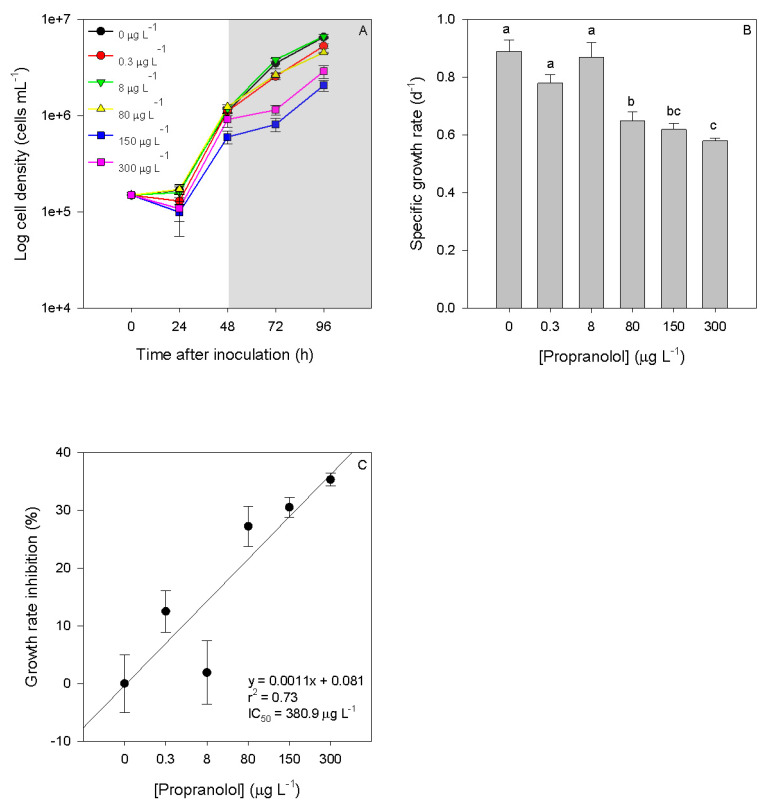
Growth curves ((**A**), grey area corresponds to the exposure period), specific growth rate (**B**) and growth inhibition percentages (**C**) of *Phaeodactylum tricornutum* cultures exposed to different propranolol concentrations (average ± standard error, *n* = 3, letters denote differences at *p* < 0.05).

Diatom growth after a propranolol 48-h exposure period was evaluated, and significant effects could be observed. At the end of the 48-h exposure-period, cultures exposed to 150 and 300 µg L^−1^ propranolol showed evident lower cell densities (Figure 1A). Additionally, Spearman correlation analysis revealed that cell density at 96 h showed a significant dose–response pattern (r^2^ = −0.79, *p* < 0.05). Cultures exposed to the propranolol concentrations above 80 µg L^−1^ showed significantly lower growth rates (Figure 1B). Considering growth inhibition after 48 h propranolol exposure (Figure 1C), the propranolol IC_50_ concentration (IC_50_ = 380.9 µg L^−1^) was calculated for the tested conditions.

### 3.2. Photobiological Traits

**Figure 2:**
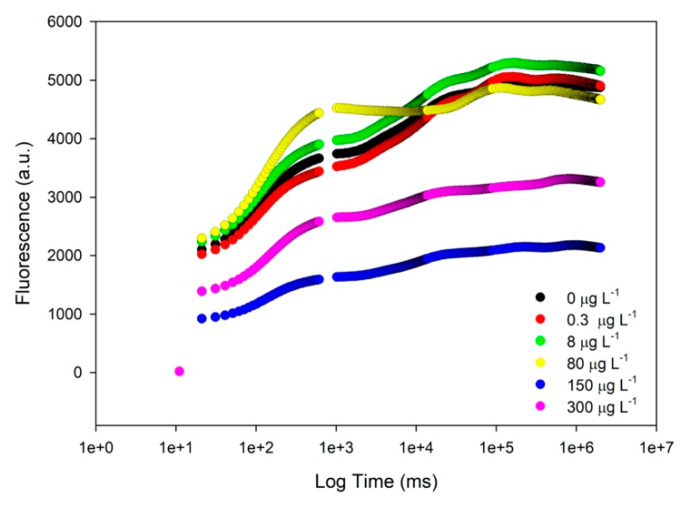
Kautsky plot curves (fluorescence in arbitrary units (a.u.)) from *Phaeodactylum tricornutum* cultures exposed to the different propranolol concentrations (average ± standard error, *n* = 3).

At the end of the exposure trials, cells were subjected to a high throughput fluorometric analysis, where several significant effects on the photochemical apparatus were detected, namely in terms of the chlorophyll a transient light curves. Observing the generated Kautsky plot curves (Figure 2) that reflect the whole photobiological performance of the cultures, again severe changes in the intensity and shape of the curves of diatoms exposed to high propranolol concentrations were observed. The curves correspondent to the control, 0.3 and 8 µg L^−1^ propranolol exposed cultures presented very similar fluorescence intensity values and a typical Kautsky curve shape. The fluorescence profile of the cultures exposed to 80 µg L^−1^ showed a more pronounced inflexion point between 500 and 1000 ms, corresponding to changes in the photobiological traits of the cultures. Likewise, exposure to the highest propranolol concentrations (150 and 300 µg L^−1^), resulted in a clear loss of the characteristic inflexion points and a severe reduction in fluorescence.

**Figure 3:**
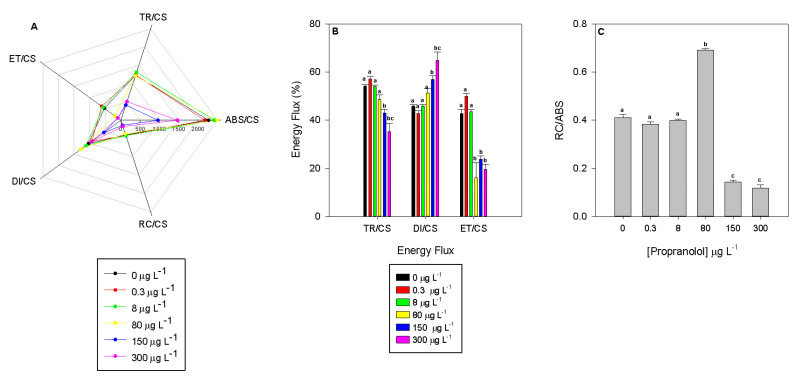
Absolute (**A**) and relative (**B**) phenomological energy fluxes (ABS/CS—absorbed energy flux per cross-section; TR/CS—trapped energy flux per cross-section; ET/CS—electron transport energy flux per cross-section; DI/CS—dissipated energy flux per cross-section and RC/CS—number of available reaction centers per cross-section) and (**C**) reaction centre density (RC/ABS) in *Phaeodactylum tricornutum* cultures exposed to different propranolol concentrations (average ± standard error, *n* = 3, letters denote differences at *p* < 0.05).

These curves translate into photobiological parameters that can be analyzed to disclose exposure effects of propranolol in the PSII photochemistry of *P. tricornutum* (Figure 3). These photobiological traits result in four principal phenomological energy fluxes: absorption (ABS/CS), trapped (TR/CS), transported (ET/CS) and dissipated (DI/CS) energy fluxes. Cultures exposed to 150 and 300 µg L^−1^ showed significantly lower energy fluxes (Figure 3A). Additionally, the absorption, trapped and transported energy fluxes showed a negative dose-dependent relationship, declining significantly with increasing propranolol exogenous concentrations (r^2^_ABS/CS_ = −0.66, r^2^_TR/CS_ = −0.83 and r^2^_ET/CS_ = −0.81, *p* < 0.05). Although this relationship was true when absolute values were used, if relative values were considered, normalizing each flux to the one that originated it, some differences ensued (Figure 3B). Considering the relative transported energy fluxes, severe depletion of its value in the cultures exposed to 80, 150 and 300 µg L^−1^ (r^2^ = −0.72, *p* < 0.05) was observed. This resulted in a proportionally higher relative dissipated energy flux in these same cultures (r^2^ = 0.93, *p* < 0.05). The significant decrease in the absorption energy flux results not only from a reduction in the RC centre density in the PSII antennae (Figure 3C, RC/ABS; r^2^ = −0.62) but also from the reduced number of oxidized reaction centers (RC/CS; r^2^ = −0.84), which inevitably cascaded down to the remaining energy fluxes (Figure 3A). This led to a severe reduction of the relative electron transport energy flux along with the exogenous propranolol concentration applied (r^2^ = −0.72, *p* < 0.05).

**Figure 4:**
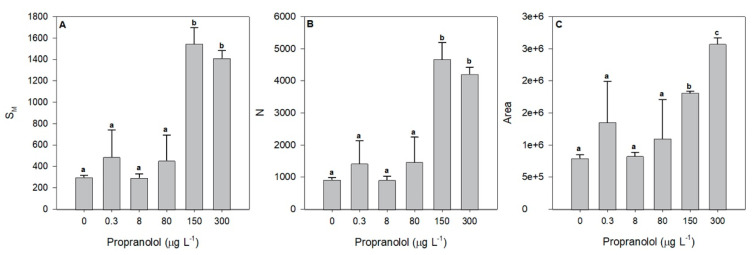
Energy required to close all RCs (S_M_, **A**), RC turnover rate (N, **B**) and the size of the quinone pool (area, **C**) in *Phaeodactylum tricornutum* cultures exposed to different propranolol concentrations (average ± standard error, *n* = 3, letters denote differences at *p* < 0.05).

The required energy to close all RCs (Figure 4A, S_M_) and its turnover rates (Figure 4B, N) also increased significantly after exposure to higher propranolol concentrations, of 150 and 300 µg L^−1^. Moreover, these parameters were shown to significantly increase with propranolol concentrations (r^2^_SM_ = 0.79 and r^2^_N_ = 0.78, *p* < 0.05). Regarding the structure and function of the electron transport chain (ETC), some changes were also observed. The size of the oxidized quinone pool available to transport electrons was drastically increased under the exposure to 150 and 300 µg L^−1^ propranolol (Figure 4C, area), showing a significant increase along the propranolol concentration applied (r^2^ = 0.72, *p* < 0.05).

**Figure 5:**
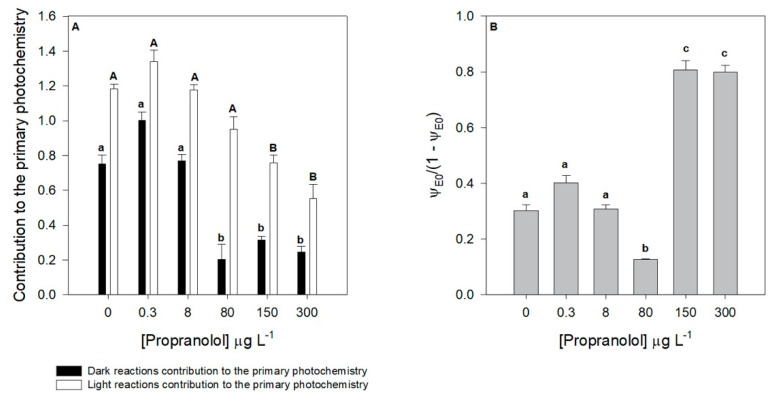
Contribution of the light and dark reactions to the primary photochemistry (**A**) and redox equilibrium constant between photosystem I and II (**B**) in *Phaeodactylum tricornutum* cultures exposed to different propranolol concentrations (average ± standard error, *n* = 3, letters denote differences at *p* < 0.05).

Considering the light and dark reactions contribution to the primary photochemistry, similar tendencies could be observed (Figure 5A). Nevertheless, some differences in both processes were evident. The contribution from the dark reaction was severely depleted in the cultures exposed to concentrations of 80 µg L^−1^ propranolol and above, having a significant dose–response tendency (r^2^ = −0.92, *p* < 0.05). Likewise, the contribution from the light reaction was affected under the exposure to 150 and 300 µg L^−1^ of propranolol. Considering both photosystems involved in the photochemical process, here analyzed by its equilibrium constant (Figure 5B), it can be observed that this ratio suffered a shift towards the PSII along the increasing propranolol exposure gradient (r^2^ = 0.73, *p* < 0.05), indicating a more severe effect at the photosystem I (PSI) level, leading to an increase of this variable in the cultures exposed to 150 and 300 µg L^−1^ of propranolol could also be observed. Additionally, a significant decrease in this equilibrium constant in the cultures exposed to 80 µg L^−1^ it was also observed.

### 3.3. Energy Allocation and Consumption

**Figure 6:**
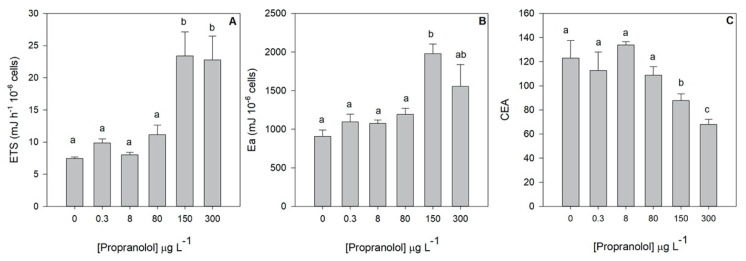
Mitochondrial electron transport system (ETS, **A**), available energy (Ea, **B**) and cellular energy allocation (CEA, **C**) in *Phaeodactylum tricornutum* cultures exposed to different propranolol concentrations (average ± standard error, *n* = 3, letters denote differences at *p* < 0.05).

Additionally, to the primary photochemistry evaluation, also the mitochondrial respiratory activity and energy reserves allocation was evaluated at the end of the propranolol exposure trial. Mitochondrial electron transport (ETS) and available energy (Ea) were significantly increased under the application of 150 and 300 μg L^−1^ propranolol (Figure 6). This increase in available energy was boosted by the significant increase of the cell protein content in exposure concentrations ranging from 80 to 300 μg L^−1^ (Table 2). In fact, this increase shows a highly significant positive trend alongside the exogenous propranolol gradient (r^2^ = 0.77, *p* < 0.05). Carbohydrates showed the inverse trend (r^2^ = −0.50, *p* < 0.05), with significantly lower concentrations in the cells exposed to 80 and 150 μg L^−1^ propranolol (Table 2). Total lipid concentrations were only found to be significantly higher in the cells exposed to 150 μg L^−1^ propranolol (Table 2). Additionally, ETS and Ea showed a significant positive correlation with the exogenous propranolol concentration (r^2^_ETS_ = 0.80, r^2^_Ea_ = 0.62, *p* < 0.05). On the other hand, cellular energy allocation (CEA) showed the inverse trend (r^2^ = −0.79, *p* < 0.05), being significantly inhibited in the cultures exposed to the highest propranolol concentrations tested (150 and 300 μg L^−1^).

### 3.4. Oxidative Stress

**Figure 7:**
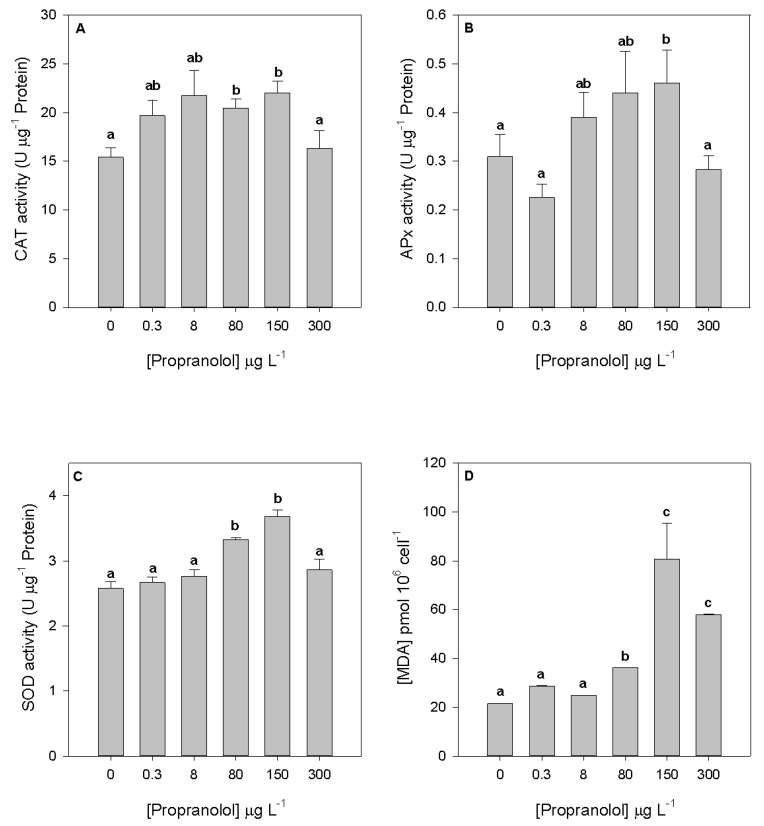
Catalase (**A**), ascorbate peroxidase (**B**), superoxide dismutase (**C**) activities and lipid peroxidation products (**D**) in *Phaeodactylum tricornutum* cultures exposed to different propranolol concentrations (average ± standard error, *n* = 3, letters denote differences at *p* < 0.05).

To evaluate potential cell damages due to oxidative stress conditions induced by propranolol exposure, several oxidative stress biomarkers were evaluated in the diatom cells exposed to the different propranolol levels. Considering the peroxidasic (CAT and APx) activities (Figure 7A,B), despite the observed increasing trend up to the 150 µg L^−1^ propranolol exposure concentration, significant changes were only detected at 80 and 150 µg L^−1^, respectively. Nevertheless, CAT and SOD activities (Figure 7C) showed a significant increase in the cultures exposed to 80 and 150 µg L^−1^ propranolol. The activity of these enzymes also showed a positive correlation with the exogenous propranolol dose applied (r^2^ = 0.67, *p* < 0.05). It is also worth noting that all enzymatic activities analyzed showed lower values at the highest propranolol concentration. Regarding MDA production derived from lipid peroxidation, significantly higher values were found in cultures exposed to 80, 150 and 300 µg L^−1^ propranolol (Figure 7D), evidencing also a positive correlation with the exogenous propranolol (r^2^ = 0.67, *p* < 0.05).

### 3.5. Fatty Acids Profile

**Figure 8:**
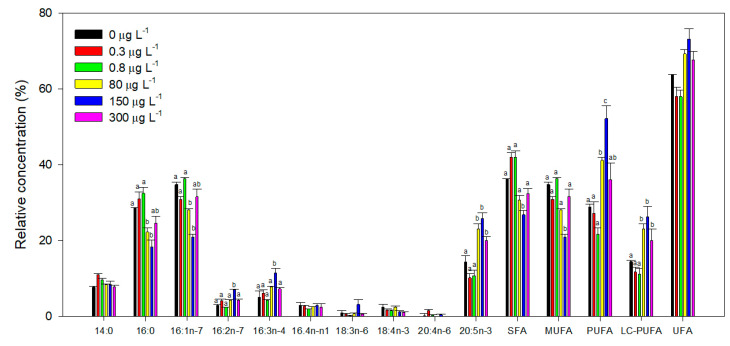
Fatty acid relative concentration (%) and saturation class abundance (%, SFA—Saturated Fatty Acids; MUFA—Monounsaturated Fatty Acids; PUFA—Polyunsaturated Fatty Acids; LC-PUFA—Long Chain Polyunsaturated Fatty Acids; UFA—Unsaturated Fatty Acids) in *Phaeodactylum tricornutum* cultures exposed to different propranolol concentrations (average, *n* = 3, letters denote differences at *p* < 0.05).

At the end of the exposure trials, cells were also evaluated concerning their total fatty acid profile, and several significant effects could be detected in this regard. When analyzed individually, some fatty acids showed significant differences in their relative abundance among treatments (Figure 8; Table S1). Fatty acids 16:2 and 16:3 showed an evident increase in cultures exposed to 150 µg L^−1^ propranolol. Additionally, the 20:5 fatty acid also increased in cells exposed to concentrations between 8 and 300 µg L^−1^ propranolol. Although no significant changes could be assessed within each of the remaining fatty acids relative concentration among different propranolol concentrations, several fatty acids revealed a dose-dependent response (Figure 8). Specifically, 16:0 and 18:4 fatty acids displayed a significant depletion along the exogenous propranolol gradient (r^2^_16:0_ = −0.55, r^2^_18:4_ = −0.50, *p* < 0.05), whereas 16:3 and 20:5 fatty acids showed a significant rise with increasing propranolol concentrations (r^2^_16:3_ = 0.50, r^2^_20:5_ = 0.58, *p* < 0.05). Regarding unsaturation classes, propranolol induced a reduction of saturated (SFA) and monounsaturated (MUFA) fatty acids at 150 µg L^−1^. In fact, SFA relative concentrations translated into a negative dose-dependent tendency along the applied propranolol gradient (r^2^ = −0.57, *p* < 0.05). Additionally, a significant increase in the polyunsaturated fatty acids (PUFAs) relative concentration in the cells exposed to 80 and 150 µg L^−1^ propranolol was also observed. Long-chain polyunsaturated fatty acids (LC-PUFAs) relative concentrations also increased at propranolol concentrations higher than 80 µg L^−1^ (Figure 8). Both PUFA and LC-PUFA cellular concentration showed a significant positive correlation with the exogenous dose applied (r^2^_PUFA_ = 0.50, r^2^_LC-PUFA_ = 0.56, *p* < 0.05). While unsaturated fatty acids (UFAs) relative concentration did not show any pairwise significant differences, a significant positive correlation could be observed with propranolol concentration present in the culture medium (r^2^ = 0.57, *p* < 0.05).

**Figure 9:**
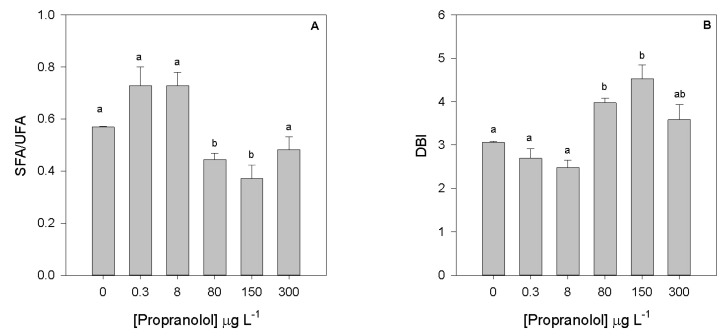
Saturated to unsaturated fatty acid ratio (**A**) and fatty acid double-bound index (**B**) in *Phaeodactylum tricornutum* cultures exposed to different propranolol concentrations (average, *n* = 3, letters denote differences at *p* < 0.05).

Contrarily, SFA/UFA ratio (Figure 9A) showed the inverse trend (r^2^ = 0.57, *p* < 0.05), with significantly lower values observed in cells exposed to 80 and 150 µg L^−1^. The DBI (Figure 9B) of cultures exposed to 80 and 150 µg L^−1^ showed a pronounced increase, and a positive correlation with the propranolol dose applied (r^2^ = 0.53, *p* < 0.05).

### 3.6. Overall Metabolic Impacts of Propranolol Exposure

**Figure 10:**
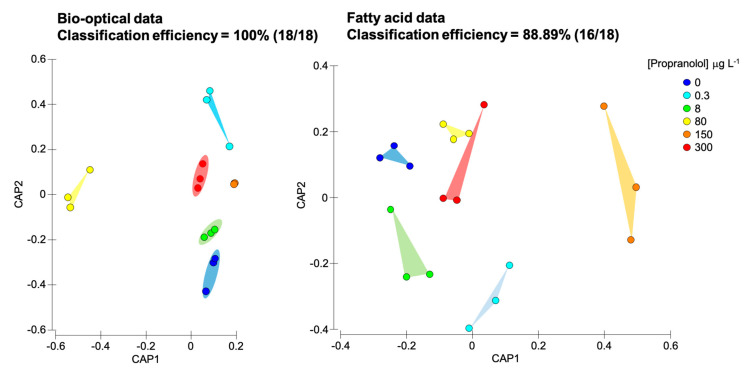
Canonical analysis of principal (CAP) components of the bio-optical and fatty acids profiles obtained from the analysis of *Phaeodactylum tricornutum* cultures exposed to the different propranolol concentrations.

To evaluate the impact of propranolol exposure on the photochemical and fatty acids metabolism, a multivariate canonical analysis was performed (Figure 10). The canonical analysis of principal components (CAPs) provides a classification efficiency of the samples according to the provided descriptors (in this case exogenous propranolol concentrations applied), having as input the variables evaluated in each metabolic compartment. In this evaluation, it is possible to perceive that both datasets efficiently separated sample groups exposed to different exogenous propranolol concentrations. The bio-optical data based-model had a classification efficiency of 100%, being an efficient descriptor of all tested concentrations and their effects on *P. tricornutum*. Regarding fatty acids profiles, the canonical classification efficiency decreased to 88.89%, due to a misclassification of two of the tested samples. Considering the classification efficiencies here addressed, the photochemical and fatty acid metabolisms changes are highly affected by propranolol exposure, presenting significant differences induced by the different propranolol exposure concentrations.

## 4. Discussion

The present work intended to investigate whether propranolol, a human pharmaceutical widely found in the aquatic environment, hinders with the energetic and lipidic metabolism of a marine diatom. Propranolol is a prototypical β-adrenoceptor antagonist applied for human cardiovascular conditions treatment [60]. The environmental propranolol concentrations range from 56 ^1^ in estuaries [16] to 590 ng L^−1^ in rivers [17], 1900 ng L^−1^ in wastewater effluents [18] and 6500 ng L^−1^ in hospital effluents [19]. According to Claessens et al. (2013), propranolol effective concentrations producing effects in 10% (EC_10_) and 50% (EC_50_) would be situated within the range of 90 to 288 µg L^−1^. In the present study, the determined IC_50_ value was above this previously reported range. The concentrations tested here included a range from environmentally detected concentrations and the abovementioned reported effective concentration ranges known to produce significant effects in *P. tricornutum* [20,41]. Nevertheless, the strain here used under the tested culture conditions showed a higher IC_50_ value (380.9 µg L^−1^). According to the results reported here, medium propranolol exposure (8 µg L^−1^) seems to stimulate the growth of *P. tricornutum*, as compared to other propranolol levels growth and concomitant with several other parameters here addressed. Previous works [37] showed that at certain non-toxic concentrations diatoms can transform and degrade aromatic compounds (as is the case of propranolol), and use the metabolization products for mixotrophic growth, increasing cell growth.

In terms of biological activity, propranolol is designed to block β-adrenergic receptors to threaten a wide number of cardiovascular conditions. Although plants and algae do not have these types of receptors, plant receptor-like kinases have similar biochemical properties and can, therefore, be targeted by this molecule [22]. CrRLK1Ls, a class of plant receptor-like kinases are linked to ROS production, and a downregulation of these receptors indicates a strategy to decrease the harmful effects of an oxidative burst, common to abiotic stress responses [22]. In plants, these PS-LRR (plant-specific leucine-rich repeat) comprising receptors are often intricate in inborn immune and developmental responses [23]. Previous studies showed that plant mutants lacking RLK exhibited lower biomass production, followed by decreased stomatal conductance and higher ROS levels, which are known to hinder photosynthetic efficiency [61,62,63]. Moreover, these mutants exhibited impaired acclimation to abiotic stress, mostly due to disrupted activity of ROS-scavenging enzymes and increased cell death [61]. Analyzing the available databases (InterProScan 5 [64], http://www.ebi.ac.uk/interpro/search/sequence-search; SMART [65], http://smart.embl-heidelberg.de/; NCBI Conserved Domain Search [66], http://www.ncbi.nlm.nih.gov/Structure/lexington/lexington.cgi?cmd=rps, accessed November 2020), the PS-LRR domains of the marine diatom *P. tricornutum* receptor-like kinases frequently presented resemblance to receptor-like kinases and receptor-like proteins (RLPs) of terrestrial plants such as CLAVATA1, GSO1 (*Arabidopsis thaliana*), Cf−2, Hcr2 (*Solanum lycopersicum*) and Xa21 (*Oryza sativa*). In our study, SOD activity increase was positively correlated with propranolol exogenous concentration. Although the activity of ROS scavenging enzymes (SOD, CAT and APX) was enhanced, this is most likely insufficient to counteract the deleterious effects of these molecules since the levels of MDA were higher in propranolol exposed cells. Contrarily to what is commonly found under other stress conditions [30,37], this oxidative burst and excessive cellular redox potential do not result from excessive energy accumulation in the photochemical apparatus. Cells exposed to increasing concentrations of propranolol appear to efficiently dissipate the excessive energy that is being absorbed by the photosystem II (PSII) and that is not being correctly directed to the electron transport chain. The interruption of the energy transduction from the PSII to the ETC increases the probability of occurrence of a possibly dangerous condition of excessive redox power increase within the photosystems, which can ultimately cause photoinhibition and D1 protein destruction and subsequent inactivation of the PSII restoration cycle and its permanent deactivation [67]. Nevertheless, the elevated amount of dissipated energy under exposure to higher propranolol concentrations could denote that the stored energy at the PS II donor side is proficiently diverted from the photosystems acting efficiently as a counteracting measure towards irreversible photoinhibition [68]. Cells appear to develop positive feedback to overcome this potential oxidative burst due to excessive intracellular free-energy levels. The RC centre density in the PSII antennae, and the number of oxidized reaction centers, showed a marked decrease, accompanied by an increase in the required energy to close all RCs, and in the RCs turnover rates. This prevents excessive photonic energy to be absorbed and that would not be used for chemical energy generation in the ETC. Previous works using *A. thaliana* mutants lacking receptor-like kinases showed an increase in photochemical quenching, similar to the one observed in the present study with increasing energy dissipation [61]. These authors suggested that the plastoquinone pool was more oxidized in the mutants lacking RLK, triggered by singlet oxygen produced in PSII or results from reduced photosynthetic antenna size, indicating reduced light-harvesting capacity. In *P. tricornutum*, propranolol-induced blockage of RLK would result in similar events, as it was observed in the cultures exposed to higher propranolol concentrations. This is concomitant with low electron transport energy fluxes, although the size of the oxidized quinone pool tends to increase along the propranolol gradient and thus, in the correct redox state, to be used as electron transporters. The maintenance of the quinone pool structure and function seems to be ensured by the increase in 16:3 fatty acid abundance under propranolol exposure. This fatty acid is highly present in plastidial galactolipids such as monogalactosyldiacylglycerol (MGDG) and digalactosyldiacylglycerol (DGDG), and its increase likely indicates an increase of these lipid classes, essential for the maintenance of the electron transport mechanisms [36]. The lipid environment adjacent to the photosystems plays an important role in these photochemical complexes’ maintenance and electron activity at the quinone level [69,70,71]. The positive correlation between DBI and the exogenous propranolol concentration seems to indicate a mechanism favoring fatty acid unsaturation, although the specific mechanism is still to be resolved. Triacylglycerols (TAG) in diatoms are typically constituted by 16:0 fatty acids [72]. In the present work, the fact that 16:0 displays a significant depletion along the exogenous propranolol gradient suggests the consumption of these storage lipids. As energy production is impaired at the PSII donor side, preventing energy production through the ETC, cells appear to be consuming TAG as an energy backup system, to maintain essential cellular functions [73]. Moreover, propranolol is known to interact directly with phospholipase D, increasing the phosphatidic acid (PA) pool [74]. This fact can be also connected to the abovementioned increase in DGDG as PA can be hydrolyzed by PA phosphatase into DGDG [74]. Additionally, PA has an essential role in cell signaling as an intracellular second messenger and can thus influence the cell response to external stress [74].

Additionally, it is worthy of notice that in the cultures exposed to 80 µg L^−1^ some photochemical traits (RC/ABS and ψ_E0_/(1 − ψ_E0_)) showed a different tendency from the observed at lower and higher concentrations, indicating a possible tipping point located between 8 and 150 µg L^−1^, where *P. tricornutum* cells respond in a non-monotonic way, that can be associated to energetic metabolism shifts [37]. Although an apparent positive feedback mechanism, activated to prevent oxidative stress induced by propranolol exposure was observed, the contribution of the dark reactions to primary photochemistry was severely impaired. This inhibition prevents the regeneration of the substrates, essential for the correct functioning of the Calvin cycle downstream the PSI [56], and thus reducing carbon fixation and oxygen production, and inevitably cell biomass production. Previous reports indicated that, in some conditions, the energy can be diverted from the ETC to the cleavage of benzenic skeletons, such as those found in propranolol structure [37]. In this case, cells undergo a shift from autotrophic to mixotrophic [75,76,77]. As already referred, in the present work, the photochemical energy trapping shows a severe depletion in favor of the increase in energy dissipation and thus, cells were unable to use this potentially diverted energy and propranolol as a substrate for cell growth, as observed in *P. tricornutum* cells exposed to bezafibrate [37].

Beyond the observed effects at the photochemical level, also the mitochondrial respiratory energy pathway showed evident signs of stress induced by propranolol. A substantial increase in the activity of the mitochondrial respiratory electron transport was observed in diatoms exposed to propranolol. One of the major consequences in terms of oxidative stress is ROS increase, due to a boosted formation and/or ineffective scavenging of these toxic molecules under stress circumstances, generated during oxidative phosphorylation [48]. A decay in CEA indicates a drop in the net energy budget and, therefore, fewer energy assigned to fundamental functions (e.g., growth) [49]. There is a significant lack of literature of the propranolol mode of action in autotrophic organisms, though heterotrophic and autotrophic model organisms are very different, in terms of mitochondrial transporters these are highly conserved between organisms, and thus some comparisons at this level can be made to disclose some of the possible mechanisms of this molecule at the mitochondrial level. In heterotrophs, propranolol is known to inhibit mitochondrial electron transport [78,79,80]. In rat cardiac tissue the NADH-oxidase, NADH-cytochrome c reductase and heart inner membrane mitochondrial transporters activity were depressed by propranolol [80]. Nevertheless, here the inverse trend was observed, which can be a positive feedback mechanism from the cells towards propranolol exposure. If both the chloroplastidial and mitochondrial electron transport chains are compared using ET/CS and ETS as respective activity proxies, an inverse correlation is evident (r^2^ = −0.72, *p* < 0.05). In energetic terms, this indicates that exposure to propranolol reduces the autotrophic energy production and increases respiratory activity. This is concomitant with the results of CEA while compared with the ET/CS, where a positive correlation can be observed (r^2^ = 0.63, *p* < 0.05), indicating that the cells are not generating energetic substrates at the same rate that are being consumed at the mitochondrial respiratory chains. Under stress and phototrophic inhibition, stressed diatoms increase their respiratory activity to obtain energy from sources other than the primary photochemistry [33], in an attempt to meet the energy needs of the cellular metabolism (using for instance carbohydrates as substrates for energy generation). Our results show a decrease in carbohydrates with increasing propranolol dosage, which may indicate that these compounds are being used for energy production. On the other hand, the boost observed in the energy allocated (Ea) is principally due to increased protein content, which can probably be linked to enzyme production such as the antioxidant stress enzyme. Considering the primary oxygen production and consumption pathways, propranolol can severely decrease diatom-driven oxygen production in marine ecosystems, not only due to reduction of the autotrophic O_2_ production but also due to the increase in the heterotrophic mitochondrial respiration.

Besides the abovementioned physiological traits, the ecotoxicological effects of propranolol exposure are also worthy of mentioning. In ecotoxicological terms, propranolol showed an IC_50_ of 380.9 µg L^−1^ under the present *P. tricornutum* culture conditions, which is above the previously reported EC_10_ and EC_50_ (90–288 µg L^−1^ respectively) reported for this species under propranolol exposure [20,41]. Nevertheless, several authors assessed IC_50_ of 1.6–7.5 mg L^−1^ using *Daphnia magna* [81,82], and IC_50_ values of 0.7–5.8 and 0.5 mg L^−1^ using green microalgae *Desmodesmus subspicatus* and *Pseudokirchneriella subcapitata,* respectively [83]. In this regard, *P. tricornutum* appears to be more sensitive to propranolol, with values up to 1000 times lower than the abovementioned organisms. Considering the physiological traits evaluated here, it becomes evident that propranolol impacts on the antioxidant enzymatic are not substantial, neither under environmental nor ecotoxicological propranolol concentrations exposure. On the other hand, considering the results from the canonical classification efficiencies, fatty acid profiles are highly affected by propranolol exposure, with an overall high degree of classification when these traits are used as a profile, instead of individually. Overall, bio-optical data shows even higher classification efficiency, indicating that the photochemical primary production metabolism is highly affected by propranolol exposure in *P. tricornutum*.

## 5. Conclusions

The presence of antihypertensive pharmaceuticals such as the β-adrenergic receptor blocker propranolol imposes a new treat to the marine environment. These molecules are designed to target specific human receptors, but their action is not limited to humans, or animals, and affect evolutionarily conserved receptors in other taxonomic groups, including algae. Using a model diatom for evaluation of the toxicological effects of these compounds, at the tested concentrations, propranolol exposure leads to impairments in the photochemical and fatty acid metabolisms, which in extreme cases may have serious impacts in the marine environment. Propranolol metabolic impacts in *P. tricornutum* led not only to a reduction of photochemical primary production but also to a significant increase in the respiratory activity of exposed diatoms, increasing O_2_ consumption, adding a supplementary factor to the depletion in diatom oxygenation capacity. At reported EC_50_ concentrations (252–329 μg L^−1^ according to [20] and 380.9 μg L^−1^ according to this study), the evaluated bio-optical and biochemical features here evaluated, namely photochemical and fatty acid metabolisms, were highly affected by propranolol exposure in the model diatom *P. tricornutum*.

## Figures and Tables

**Table 1:** Summary of fluorometric analysis parameters and their description.

OJIP-Test	Parameter Description
Area	Corresponds to the oxidized quinone pool size available for reduction and is a function of the area above the Kautsky plot
N	Reaction center turnover rate
S_M_	Corresponds to the energy needed to close all reaction centers
P_G_	Grouping probability between the two PSII units
ABS/CS	Absorbed energy flux per cross-section
TR/CS	Trapped energy flux per cross-section
ET/CS	Electron transport energy flux per cross-section
DI/CS	Dissipated energy flux per cross-section
RC/CS	Number of available reaction centers per cross-section
%TR	Relative trapped energy flux per cross-section (%TR = TR/CS/ABS/CS)
%ET	Relative electron transport energy flux per cross-section (%ET = ET/CS/TR/CS)
%DI	Relative dissipated energy flux per cross-section (%DI = DI/CS/ABS/CS)
TR_0_/DI_0_	Contribution or partial performance due to the light reactions for primary photochemistry
φ_o_/(1 − φ_o_)	Contribution or partial performance due to the dark reactions for primary photochemistry
ψ_E0_/(1 − ψ_E0_)	Equilibrium constant for the redox reactions between PS II and PS I
RC/ABS	Reaction center II density within the antenna chlorophyll bed of PS II

**Table 2:** Total proteins, carbohydrates and lipids *Phaeodactylum tricornutum* cultures exposed to different propranolol concentrations (average ± standard error, *n* = 3, letters denote differences at *p* < 0.05).

[Propranolol] μg L^−1^	Proteins (mJ 10^−6^ Cells)	Carbohydrates (mJ 10^−6^ Cells)	Lipids (mJ 10^−6^ Cells)
0	183.4 ± 22.7 ^a^	106.7 ± 15.5 ^a^	618.0 ± 43.8 ^a^
0.3	277.1 ± 47.3 ^a^	62.4 ± 19.7 ^ab^	756.4 ± 41.3 ^a^
8	239.6 ± 25.0 ^a^	111.3 ± 5.0 ^a^	723.1 ± 28.8 ^a^
80	422.9 ± 33.7 ^b^	33.3 ± 7.4 ^b^	734.6 ± 59.5 ^a^
150	515.4 ± 49.1 ^b^	6.5 ± 1.8 ^c^	1458.6 ± 73.5 ^b^
300	571.2 ± 139.46 ^b^	51.2 ± 14.3 ^ab^	933.3 ± 154.9 ^ab^

## Supplementary Materials

The following are available online at https://www.mdpi.com/2079-7737/9/12/478/s1, Table S1: Fatty acid and saturation classes relative concentrations in *Phaeodactylum tricornutum* cultures exposed to different propranolol concentrations (average ± standard error, *n* = 3).
