# Supplementary material for: Effects of Propranolol on Growth, Lipids and Energy Metabolism and Oxidative Stress Response of Phaeodactylum tricornutum
Source: Biology (Basel). 2020 Dec 18;9(12):478. doi: 10.3390/biology9120478 (PMC7766914; doi:10.3390/biology9120478)
Supplement: Supplementary file 1 [file biology-09-00478-s001.pdf]

**Table S1.** Fatty acid and saturation classes relative concentrations in *Phaeodactylum tricornutum* cultures exposed to different propranolol concentrations (average ± standard error, n = 3).

| | 0 µg L-1 | 0.3 µg L-1 | 8 µg L-1 | 80 µg L-1 | 150 µg L-1 | 300 µg L-1 |
|---|---|---|---|---|---|---|
| **14:0** | 7.76 ± 0.40 | 11.01 ± 0.66 | 9.59 ± 0.84 | 8.45 ± 0.23 | 8.50 ± 1.55 | 7.85 ± 0.71 |
| **16:0** | 28.52 ± 0.36 | 30.94 ± 3.38 | 32.44 ± 2.72 | 22.28 ± 1.84 | 18.34 ± 3.15 | 24.55 ± 3.35 |
| **16:1 n-7** | 34.82 ± 1.20 | 30.91 ± 1.33 | 36.34 ± 0.46 | 28.10 ± 0.46 | 20.96 ± 1.33 | 31.55 ± 3.46 |
| **16:2 n-7** | 2.96 ± 0.46 | 4.16 ± 0.64 | 2.43 ± 0.08 | 4.32 ± 0.30 | 7.13 ± 0.05 | 4.25 ± 0.58 |
| **16:3 n-4** | 5.04 ± 0.23 | 6.089 ± 1.59 | 4.36 ± 0.13 | 7.82 ± 0.30 | 11.49 ± 2.02 | 7.33 ± 0.64 |
| **16:4 n-1** | 2.94 ± 0.42 | 2.73 ± 0.49 | 1.95 ± 0.24 | 2.63 ± 0.10 | 2.96 ± 0.70 | 2.56 ± 1.34 |
| **18:3 n-6** | 0.92 ± 0.25 | 0.69 ± 0.24 | 0.23 ± 0.18 | 0.74 ± 0.43 | 3.16 ± 2.29 | 0.68 ± 0.21 |
| **18:4 n-3** | 2.55 ± 0.41 | 1.76 ± 0.33 | 1.51 ± 0.40 | 2.55 ± 0.50 | 1.24 ± 0.62 | 1.15 ± 0.22 |
| **20:4 n-6** | 0.14 ± 0.12 | 1.50 ± 0.52 | 0.40 ± 0.26 | 0.00 ± 0.00 | 0.48 ± 0.36 | 0.02 ± 0.03 |
| **20:5 n-3** | 14.36 ± 0.80 | 10.21 ± 1.93 | 10.75 ± 2.58 | 23.11 ± 2.29 | 25.74 ± 4.54 | 20.07 ± 5.20 |
| **SFA** | 36.27 ± 0.06 | 41.95 ± 2.33 | 42.02 ± 1.68 | 30.73 ± 1.14 | 26.84 ± 2.71 | 32.39 ± 2.33 |
| **MUFA** | 34.82 ± 0.69 | 30.91 ± 0.77 | 36.34 ± 0.27 | 28.10 ± 0.27 | 20.96 ± 0.77 | 31.55 ± 2.00 |
| **PUFA** | 28.90 ± 0.64 | 27.14 ± 3.09 | 21-64 ± 1.73 | 41.17 ± 0.89 | 52.20 ± 3.43 | 36.06 ± 4.33 |
| **LC-PUFA** | 14.50 ± 0.39 | 11.71 ± 1.28 | 11.15 ± 1.47 | 23.11 ± 1.32 | 26.22 ± 2.81 | 20.08 ± 2.99 |
| **UFA** | 63.73 ± 0.06 | 58,05 ± 2.33 | 57.98 ± 1.68 | 69.27 ± 1.14 | 73.16 ± 2.71 | 67.61 ± 2.33 |

**Publisher's Note:** MDPI stays neutral with regard to jurisdictional claims in published maps and institutional affiliations.

© 2020 by the authors. Licensee MDPI, Basel, Switzerland. This article is an open access article distributed under the terms and conditions of the Creative Commons Attribution (CC BY) license (http://creativecommons.org/licenses/by/4.0/).
